# Bis[μ-4-(2-oxidobenzyl­idene)thio­semi­carbazidato-κ^4^ *S*,*N* ^1^,*O*:*O*]bis­[(pyridine-κ*N*)zinc]

**DOI:** 10.1107/S160053681105522X

**Published:** 2012-01-07

**Authors:** Reza Takjoo, Grzegorz Dutkiewicz, Mehdi Ahmadi, Maciej Kubicki

**Affiliations:** a Department of Chemistry, School of Sciences, Ferdowsi University of Mashhad, Mashhad 91775–1436, Iran; b Department of Chemistry, Adam Mickiewicz University, Grunwaldzka 6, 60–780 Poznań, Poland; c Department of Chemistry, Payame Noor University (PNU), Mashhad, Iran

## Abstract

In the title compound, [Zn_2_(C_8_H_7_N_3_OS)_2_(C_5_H_5_N)_2_], the Zn_2_O_2_ ring has a flattened roof shape, with the roof angle equal to 10.10 (6)°. The thio­semicarbazones act as tridentate ligands to one Zn^II^ atom, with the O atoms additionally in bridging positions to the second Zn^II^ atom. Both Zn^II^ atoms are five-coordinated; the coordination polyhedra are close to square pyramids, with the pyridine N atoms at apical positions. Two inter­molecular N—H⋯N and one relatively weak N—H⋯S hydrogen bond, together with C—H⋯S weak inter­actions, connect the mol­ecules into a three-dimensional network.

## Related literature

For thio­semicarbazones and their biological activity, see: Alomar *et al.* (2009 ▶); Geweely (2009 ▶); Hakimi *et al.* (2010 ▶); Hellmich *et al.* (2004 ▶); Joseph *et al.* (2004 ▶); Latheef *et al.* (2007 ▶); For background to the Cambridge Structural Database, see: Allen (2002 ▶). For similar Zn complexes, see: Cui & Hu (1994 ▶); Ma *et al.* (1996 ▶).
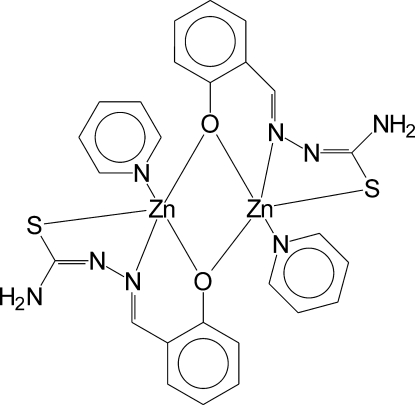


## Experimental

### 

#### Crystal data

[Zn_2_(C_8_H_7_N_3_OS)_2_(C_5_H_5_N)_2_]
*M* *_r_* = 675.45
Monoclinic, 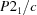

*a* = 10.2641 (3) Å
*b* = 17.3160 (6) Å
*c* = 16.6473 (5) Å
β = 104.706 (3)°
*V* = 2861.85 (16) Å^3^
*Z* = 4
Cu *K*α radiation
μ = 3.76 mm^−1^
*T* = 295 K
0.30 × 0.15 × 0.10 mm

#### Data collection

Agilent SuperNova Single source at offset Atlas diffractometer
Absorption correction: multi-scan (*CrysAlis PRO*; Agilent, 2011 ▶) *T*_min_ = 0.82, *T*_max_ = 1.00
10543 measured reflections
5646 independent reflections
4967 reflections with *I* > 2σ(*I*)
*R*_int_ = 0.024

#### Refinement

*R*[*F* ^2^ > 2σ(*F* ^2^)] = 0.031
*wR*(*F* ^2^) = 0.082
*S* = 1.05
5646 reflections
377 parameters
H atoms treated by a mixture of independent and constrained refinement
Δρ_max_ = 0.30 e Å^−3^
Δρ_min_ = −0.41 e Å^−3^

### 

Data collection: *CrysAlis PRO* (Agilent, 2011 ▶); cell refinement: *CrysAlis PRO*; data reduction: *CrysAlis PRO*; program(s) used to solve structure: *SIR92* (Altomare *et al.*, 1993 ▶); program(s) used to refine structure: *SHELXL97* (Sheldrick, 2008 ▶); molecular graphics: *SHELXTL* (Sheldrick, 2008 ▶) and *Mercury* (Macrae *et al.*, 2008 ▶); software used to prepare material for publication: *SHELXL97*.

## Supplementary Material

Crystal structure: contains datablock(s) I, global. DOI: 10.1107/S160053681105522X/rk2324sup1.cif

Structure factors: contains datablock(s) I. DOI: 10.1107/S160053681105522X/rk2324Isup2.hkl

Additional supplementary materials:  crystallographic information; 3D view; checkCIF report

## Figures and Tables

**Table 1:** Hydrogen-bond geometry (Å, °)

*D*—H⋯*A*	*D*—H	H⋯*A*	*D*⋯*A*	*D*—H⋯*A*
N12*A*—H12*B*⋯N9*B*^i^	0.80 (5)	2.40 (5)	3.195 (4)	174 (6)
N12*B*—H12*C*⋯S11*A*^ii^	0.77 (3)	2.74 (3)	3.510 (3)	177 (3)
N12*B*—H12*D*⋯N9*A*^iii^	0.88 (3)	2.14 (3)	3.012 (3)	173 (3)
C17*B*—H17*B*⋯S11*A*^iv^	0.93	2.91	3.772 (3)	156

Symmetry codes: (i) 
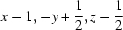
; (ii) 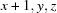; (iii) 
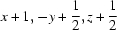
; (iv) 
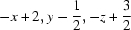
.

**Fig. 1.**
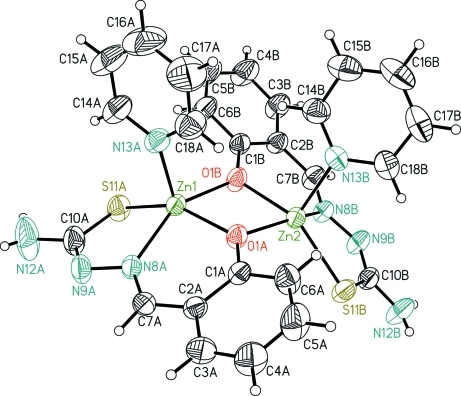
Molecular complex with tha atom numbering scheme. Displacement ellipsoids are presented at 50% probability level. Hydrogen atoms are depicted as a small spheres with arbitrary radii.

**Fig. 2.**
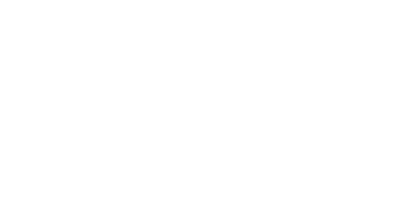
The packing diagram of title compound. View along c–direction. Hydrogen bonds are shown as dashed lines.

## supplementary crystallographic information

### Comment

Thiosemicarbazones occupy important class of *N*,*S*–donor ligands due to their great versatility (*e.g.*, Alomar *et al.*, 2009). Their complexes with transition metals have been subject of considerable interest because of their chemical and biological properties. The most important biological activities are antiviral, antifungal, antibacterial, antitumor, anticancerogenic and insulinmimetic properties (Hakimi *et al.*, 2010).

The biological activity is due to the ability to form tridentate chelates with metal ions bonding through oxygen, nitrogen and sulfur atoms (Joseph *et al.*, 2004). Zinc is essential ion to play an important role in various biological systems and may be its presence in certain metalloenzymes (Hellmich *et al.*, 2004). The Zn(II) ion has been found to be of catalytic importance in enzymatic reactions (Latheef *et al.*, 2007). The enhancement of antitumor activity of some thiosemicarbazones in the presence of Zn(II) ions has been reported (Geweely, 2009).

Herein we report the synthesis and crystal structure of a new Zn(II) complex, bis((µ^2^–salicylidenealdiminato–*N*–thiosemicarbazono–*O*,*O*,*S*,*N*)–(pyridine–*N*)–Zn(II) (Scheme 1). Some crystal structures of similar dinuclear Zn(II) complexes have been reported earlier, *e.g.* (µ_2_–hydroxo)–(µ_2_–2,6–diformyl–4–methylphenolato– bis(thiosemicarbazone))–dipyridyl–di–zinc pyridine solvate (Ma *et al.*, 1996), or bis((µ_2_–6–methoxysalicylidenealdiminato–*N*–thiosemicarbazono– *O*,*O*,*S*,*N*)–(dimethylformamide–O)–Zn(II)) (Cui & Hu, 1994).

Each of the thiosemicarbazone fragments acts as a tetradentate ligand, with oxygen atoms in bridging positions. Both Zn atoms are 5–coordinated with the coordination scheme close to square pyramid, the pyridine nitrogen atoms occupy apical positions. The four base atoms are coplanar within 0.0192 (9)Å (around Zn1) and 0.0532 (10)Å (Zn2), while the Zn and pyridine N atoms are out of these planes by 0.5273 (8)Å and -2.612 (2)Å (Zn1) and by 0.5023 (8)Å and 2.586 (2)Å (Zn2). The two apical pyridine fragments make dihedral angle of only 19.91 (8)°. The Zn_2_O_2_ ring has a flattened roof shape, with the roof angle (defined as the dihedral angle between two ZnO_2_ planes) equal to 10.10 (6)°. This value is close to the mean value found for 752 fragments from the Cambridge Structural Database (Allen, 2002), of 12°. It might be noted however that in the majority of these complexes the ZnOZnO fragment is planar due to its symmetry.

Two thiosemicarbazone molecules have very similar geometries, with elongated C—O and C—S bonds, due to involvement of the heteroatoms in the coordination. The chain fragments are in extended conformation, the whole chains are approximately - within 0.131 (2)Å and 0.0495 (16)Å - planar, and their mean planes are not far from coplanarity with the ring, the dihedral angles between the mean planes are 14.62 (12)° and 16.45 (8)°.

In the crystal structure there are two classical intermolecular N—H···N and one N—H···S relatively weak hydrogen bonds, which together with non–classical C—H···S weak interaction, connect molecules into three–dimensional network.

### Experimental

To a solution of the Zn(O*Ac*)_2_×2H_2_O (0.22 g, 1.0 mmol) in boiling 10 ml of ethanol was added a boiling solution of the 2–(2–hydroxybenzylidene)hydrazinecarbothioamide (0.20 g, 1.0 mmol) in the same solvent (10 ml) and pyridine (0.12 g, 1.5 mmol). The mixture was heated on a water bath for 1 h and left to stand for 3 days when the complexes generally crystallized from the reaction mixture. The products were filtered, washed with ethanol and dried in air.

### Refinement

The hydrogen atoms from NH_2_–groups were found in difference Fourier map and freely refined. All other hydrogen atoms were generated geometrically and refined as a riding model with their *U*_iso_(H) = 1.2*U*_eq_(C).

### Crystal data

**Table:**

[Zn_2_(C_8_H_7_N_3_OS)_2_(C_5_H_5_N)_2_]	*F*(000) = 1376
*M**_r_* = 675.45	*D*_x_ = 1.568 Mg m^−^^3^
Monoclinic, *P*2_1_/*c*	Cu *K*α radiation, λ = 1.54178 Å
Hall symbol: -P 2ybc	Cell parameters from 3324 reflections
*a* = 10.2641 (3) Å	θ = 3–27°
*b* = 17.3160 (6) Å	µ = 3.76 mm^−^^1^
*c* = 16.6473 (5) Å	*T* = 295 K
β = 104.706 (3)°	Block, colourless
*V* = 2861.85 (16) Å^3^	0.3 × 0.15 × 0.1 mm
*Z* = 4	

### Data collection

**Table:**

Agilent SuperNova Single source at offset Atlas diffractometer	5646 independent reflections
Radiation source: SuperNova X–ray Source	4967 reflections with *I* > 2σ(*I*)
mirror	*R*_int_ = 0.024
Detector resolution: 10.5357 pixels mm^-1^	θ_max_ = 75.5°, θ_min_ = 3.8°
ω scan	*h* = −12→12
Absorption correction: multi-scan (*CrysAlis PRO*; Agilent, 2011)	*k* = −21→21
*T*_min_ = 0.82, *T*_max_ = 1.00	*l* = −20→15
10543 measured reflections	

### Refinement

**Table:**

Refinement on *F*^2^	Primary atom site location: structure-invariant direct methods
Least-squares matrix: full	Secondary atom site location: difference Fourier map
*R*[*F*^2^ > 2σ(*F*^2^)] = 0.031	Hydrogen site location: inferred from neighbouring sites
*wR*(*F*^2^) = 0.082	H atoms treated by a mixture of independent and constrained refinement
*S* = 1.05	*w* = 1/[σ^2^(*F*_o_^2^) + (0.0412*P*)^2^ + 0.4387*P*] where *P* = (*F*_o_^2^ + 2*F*_c_^2^)/3
5646 reflections	(Δ/σ)_max_ = 0.001
377 parameters	Δρ_max_ = 0.30 e Å^−^^3^
0 restraints	Δρ_min_ = −0.41 e Å^−^^3^

### Special details

**Table:**

Geometry. All s.u.'s (except the s.u. in the dihedral angle between two l.s. planes) are estimated using the full covariance matrix. The cell s.u.'s are taken into account individually in the estimation of s.u.'s in distances, angles and torsion angles; correlations between s.u.'s in cell parameters are only used when they are defined by crystal symmetry. An approximate (isotropic) treatment of cell s.u.'s is used for estimating s.u.'s involving l.s. planes.
Refinement. Refinement of *F*^2^ against ALL reflections. The weighted *R*–factor *wR* and goodness of fit *S* are based on *F*^2^, conventional *R*–factors *R* are based on *F*, with *F* set to zero for negative *F*^2^. The threshold expression of *F*^2^ > σ(*F*^2^) is used only for calculating *R*–factors(gt) *etc*. and is not relevant to the choice of reflections for refinement. *R*–factors based on *F*^2^ are statistically about twice as large as those based on *F*, and *R*–factors based on ALL data will be even larger.

### Fractional atomic coordinates and isotropic or equivalent isotropic displacement parameters (Å^2^)

**Table:**

	*x*	*y*	*z*	*U*_iso_*/*U*_eq_	
Zn1	0.69348 (3)	0.151423 (17)	0.675727 (17)	0.03492 (9)	
Zn2	0.99909 (3)	0.106406 (16)	0.765896 (16)	0.03350 (8)	
O1A	0.87115 (14)	0.12696 (9)	0.64931 (8)	0.0375 (3)	
C1A	0.9113 (2)	0.13850 (12)	0.57968 (12)	0.0342 (4)	
C6A	1.0204 (3)	0.09720 (16)	0.56694 (15)	0.0502 (6)	
H6A	1.0612	0.0602	0.6057	0.060*	
C5A	1.0698 (3)	0.10973 (19)	0.49806 (17)	0.0638 (8)	
H5A	1.1445	0.0823	0.4917	0.077*	
C4A	1.0085 (3)	0.16296 (19)	0.43875 (17)	0.0637 (8)	
H4A	1.0422	0.1721	0.3928	0.076*	
C3A	0.8978 (3)	0.20210 (17)	0.44817 (15)	0.0514 (6)	
H3A	0.8554	0.2367	0.4071	0.062*	
C2A	0.8456 (2)	0.19183 (13)	0.51808 (12)	0.0357 (4)	
C7A	0.7266 (2)	0.23507 (13)	0.51998 (13)	0.0385 (5)	
H7A	0.6974	0.2720	0.4788	0.046*	
N8A	0.65690 (17)	0.22690 (11)	0.57391 (11)	0.0371 (4)	
N9A	0.5388 (2)	0.26953 (14)	0.55813 (13)	0.0520 (5)	
C10A	0.4838 (2)	0.27287 (17)	0.62098 (16)	0.0543 (6)	
S11A	0.55217 (6)	0.23729 (4)	0.72104 (4)	0.04851 (15)	
N12A	0.3637 (3)	0.3092 (3)	0.6074 (2)	0.0974 (13)	
H12A	0.323 (5)	0.304 (3)	0.645 (3)	0.120 (16)*	
H12B	0.322 (5)	0.313 (3)	0.560 (3)	0.15 (2)*	
N13A	0.58207 (19)	0.05185 (12)	0.63312 (11)	0.0442 (4)	
C14A	0.4532 (3)	0.04424 (18)	0.63634 (17)	0.0598 (7)	
H14A	0.4076	0.0872	0.6488	0.072*	
C15A	0.3874 (3)	−0.0251 (2)	0.6217 (2)	0.0808 (10)	
H15A	0.2980	−0.0292	0.6239	0.097*	
C16A	0.4555 (4)	−0.0887 (2)	0.6037 (2)	0.0929 (13)	
H16A	0.4132	−0.1366	0.5953	0.112*	
C17A	0.5849 (4)	−0.0810 (2)	0.5984 (2)	0.0856 (11)	
H17A	0.6321	−0.1231	0.5856	0.103*	
C18A	0.6442 (3)	−0.00951 (17)	0.61249 (17)	0.0591 (7)	
H18A	0.7318	−0.0038	0.6074	0.071*	
O1B	0.81432 (14)	0.11522 (10)	0.78830 (9)	0.0421 (4)	
C1B	0.7831 (2)	0.09209 (13)	0.85795 (13)	0.0371 (4)	
C6B	0.6525 (2)	0.06964 (17)	0.85587 (15)	0.0527 (6)	
H6B	0.5868	0.0725	0.8059	0.063*	
C5B	0.6168 (3)	0.0432 (2)	0.92560 (17)	0.0659 (8)	
H5B	0.5286	0.0279	0.9219	0.079*	
C4B	0.7121 (3)	0.0395 (2)	1.00065 (17)	0.0671 (9)	
H4B	0.6894	0.0211	1.0478	0.081*	
C3B	0.8406 (3)	0.06347 (18)	1.00472 (15)	0.0562 (7)	
H3B	0.9042	0.0619	1.0557	0.067*	
C2B	0.8803 (2)	0.09033 (14)	0.93500 (13)	0.0387 (5)	
C7B	1.0179 (2)	0.11677 (15)	0.94866 (14)	0.0441 (5)	
H7B	1.0676	0.1225	1.0034	0.053*	
N8B	1.07691 (17)	0.13298 (11)	0.89124 (11)	0.0373 (4)	
N9B	1.20725 (18)	0.16078 (12)	0.91816 (12)	0.0444 (4)	
C10B	1.2639 (2)	0.17693 (13)	0.85755 (14)	0.0394 (5)	
S11B	1.19620 (5)	0.16312 (4)	0.75090 (3)	0.04356 (14)	
N12B	1.3873 (2)	0.20863 (17)	0.87933 (16)	0.0588 (6)	
H12C	1.425 (3)	0.2133 (17)	0.8447 (18)	0.056 (9)*	
H12D	1.427 (3)	0.2123 (18)	0.9327 (19)	0.065 (9)*	
N13B	1.02303 (19)	−0.01384 (11)	0.76976 (11)	0.0408 (4)	
C14B	0.9228 (3)	−0.06138 (15)	0.77443 (16)	0.0510 (6)	
H14B	0.8382	−0.0406	0.7717	0.061*	
C15B	0.9403 (4)	−0.14009 (16)	0.78314 (19)	0.0645 (8)	
H15B	0.8681	−0.1719	0.7850	0.077*	
C16B	1.0642 (4)	−0.17070 (17)	0.78893 (19)	0.0712 (9)	
H16B	1.0782	−0.2236	0.7961	0.085*	
C17B	1.1681 (3)	−0.12304 (17)	0.78416 (19)	0.0678 (8)	
H17B	1.2534	−0.1430	0.7874	0.081*	
C18B	1.1437 (3)	−0.04431 (15)	0.77445 (15)	0.0514 (6)	
H18B	1.2142	−0.0117	0.7711	0.062*	

### Atomic displacement parameters (Å^2^)

**Table:**

	*U*^11^	*U*^22^	*U*^33^	*U*^12^	*U*^13^	*U*^23^
Zn1	0.02750 (14)	0.04156 (17)	0.03582 (15)	−0.00054 (11)	0.00824 (11)	0.00450 (11)
Zn2	0.02757 (14)	0.03770 (16)	0.03524 (15)	0.00084 (10)	0.00802 (10)	0.00295 (11)
O1A	0.0298 (7)	0.0517 (9)	0.0319 (7)	0.0043 (6)	0.0096 (5)	0.0079 (6)
C1A	0.0303 (10)	0.0404 (11)	0.0324 (10)	−0.0026 (8)	0.0091 (8)	−0.0011 (8)
C6A	0.0496 (13)	0.0609 (16)	0.0434 (12)	0.0145 (12)	0.0180 (10)	0.0040 (11)
C5A	0.0580 (16)	0.090 (2)	0.0519 (15)	0.0210 (15)	0.0290 (13)	−0.0017 (15)
C4A	0.0638 (17)	0.092 (2)	0.0447 (14)	0.0105 (15)	0.0316 (13)	0.0073 (14)
C3A	0.0545 (14)	0.0647 (16)	0.0390 (12)	0.0003 (12)	0.0193 (10)	0.0099 (11)
C2A	0.0339 (10)	0.0402 (11)	0.0335 (10)	−0.0043 (8)	0.0094 (8)	0.0016 (9)
C7A	0.0366 (11)	0.0441 (12)	0.0337 (10)	−0.0013 (9)	0.0068 (8)	0.0066 (9)
N8A	0.0285 (8)	0.0443 (10)	0.0375 (9)	0.0041 (7)	0.0069 (7)	0.0049 (8)
N9A	0.0368 (10)	0.0699 (14)	0.0503 (11)	0.0191 (10)	0.0126 (8)	0.0167 (10)
C10A	0.0385 (12)	0.0679 (17)	0.0583 (15)	0.0175 (12)	0.0159 (11)	0.0107 (13)
S11A	0.0420 (3)	0.0618 (4)	0.0461 (3)	0.0088 (3)	0.0193 (2)	0.0043 (3)
N12A	0.0647 (18)	0.162 (4)	0.073 (2)	0.063 (2)	0.0314 (16)	0.033 (2)
N13A	0.0395 (10)	0.0496 (11)	0.0419 (10)	−0.0086 (8)	0.0072 (8)	0.0003 (9)
C14A	0.0445 (14)	0.0732 (19)	0.0608 (16)	−0.0159 (13)	0.0117 (12)	−0.0041 (14)
C15A	0.0618 (19)	0.099 (3)	0.079 (2)	−0.0414 (19)	0.0130 (16)	−0.011 (2)
C16A	0.103 (3)	0.075 (2)	0.091 (3)	−0.051 (2)	0.008 (2)	−0.015 (2)
C17A	0.097 (3)	0.0586 (19)	0.094 (3)	−0.0134 (19)	0.012 (2)	−0.0234 (18)
C18A	0.0580 (16)	0.0554 (16)	0.0625 (16)	−0.0080 (13)	0.0128 (13)	−0.0090 (13)
O1B	0.0288 (7)	0.0654 (11)	0.0330 (7)	0.0019 (7)	0.0093 (6)	0.0104 (7)
C1B	0.0328 (10)	0.0458 (12)	0.0356 (10)	0.0008 (9)	0.0141 (8)	0.0051 (9)
C6B	0.0360 (12)	0.0794 (19)	0.0418 (12)	−0.0123 (12)	0.0081 (9)	0.0078 (12)
C5B	0.0415 (13)	0.103 (2)	0.0559 (15)	−0.0213 (15)	0.0174 (11)	0.0111 (16)
C4B	0.0536 (16)	0.107 (3)	0.0452 (14)	−0.0157 (16)	0.0207 (12)	0.0176 (15)
C3B	0.0437 (13)	0.088 (2)	0.0365 (12)	−0.0091 (13)	0.0093 (10)	0.0126 (13)
C2B	0.0351 (11)	0.0476 (12)	0.0352 (10)	−0.0025 (9)	0.0122 (8)	0.0014 (9)
C7B	0.0365 (11)	0.0619 (15)	0.0331 (11)	−0.0067 (10)	0.0073 (8)	−0.0006 (10)
N8B	0.0294 (8)	0.0444 (10)	0.0383 (9)	−0.0036 (7)	0.0092 (7)	−0.0019 (8)
N9B	0.0314 (9)	0.0592 (12)	0.0426 (10)	−0.0099 (8)	0.0091 (7)	−0.0058 (9)
C10B	0.0297 (10)	0.0423 (12)	0.0460 (12)	−0.0033 (9)	0.0090 (8)	−0.0017 (9)
S11B	0.0340 (3)	0.0560 (3)	0.0419 (3)	−0.0073 (2)	0.0119 (2)	0.0032 (2)
N12B	0.0378 (11)	0.0901 (19)	0.0503 (13)	−0.0206 (11)	0.0144 (10)	−0.0047 (13)
N13B	0.0441 (10)	0.0380 (10)	0.0388 (10)	0.0048 (8)	0.0078 (7)	0.0041 (8)
C14B	0.0553 (14)	0.0428 (13)	0.0532 (14)	−0.0043 (11)	0.0108 (11)	−0.0002 (11)
C15B	0.083 (2)	0.0447 (15)	0.0631 (17)	−0.0107 (14)	0.0144 (15)	0.0017 (13)
C16B	0.104 (3)	0.0379 (14)	0.0656 (19)	0.0098 (16)	0.0111 (17)	0.0048 (13)
C17B	0.075 (2)	0.0560 (17)	0.0676 (18)	0.0272 (16)	0.0085 (15)	0.0019 (14)
C18B	0.0511 (14)	0.0506 (14)	0.0514 (14)	0.0103 (11)	0.0110 (11)	0.0043 (11)

### Geometric parameters (Å, °)

**Table:**

Zn1—O1A	2.0263 (14)	C16A—C17A	1.360 (6)
Zn1—O1B	2.0647 (14)	C16A—H16A	0.9300
Zn1—N13A	2.0909 (19)	C17A—C18A	1.373 (4)
Zn1—N8A	2.0974 (18)	C17A—H17A	0.9300
Zn1—S11A	2.3322 (6)	C18A—H18A	0.9300
Zn2—O1B	2.0294 (15)	O1B—C1B	1.340 (2)
Zn2—O1A	2.0795 (14)	C1B—C6B	1.388 (3)
Zn2—N8B	2.0875 (18)	C1B—C2B	1.411 (3)
Zn2—N13B	2.0957 (19)	C6B—C5B	1.381 (3)
Zn2—S11B	2.3186 (6)	C6B—H6B	0.9300
O1A—C1A	1.340 (2)	C5B—C4B	1.379 (4)
C1A—C6A	1.390 (3)	C5B—H5B	0.9300
C1A—C2A	1.417 (3)	C4B—C3B	1.368 (4)
C6A—C5A	1.384 (3)	C4B—H4B	0.9300
C6A—H6A	0.9300	C3B—C2B	1.403 (3)
C5A—C4A	1.380 (4)	C3B—H3B	0.9300
C5A—H5A	0.9300	C2B—C7B	1.446 (3)
C4A—C3A	1.366 (4)	C7B—N8B	1.286 (3)
C4A—H4A	0.9300	C7B—H7B	0.9300
C3A—C2A	1.410 (3)	N8B—N9B	1.385 (2)
C3A—H3A	0.9300	N9B—C10B	1.316 (3)
C2A—C7A	1.440 (3)	C10B—N12B	1.343 (3)
C7A—N8A	1.290 (3)	C10B—S11B	1.751 (2)
C7A—H7A	0.9300	N12B—H12C	0.77 (3)
N8A—N9A	1.386 (2)	N12B—H12D	0.88 (3)
N9A—C10A	1.310 (3)	N13B—C18B	1.330 (3)
C10A—N12A	1.351 (3)	N13B—C14B	1.335 (3)
C10A—S11A	1.747 (3)	C14B—C15B	1.378 (4)
N12A—H12A	0.84 (4)	C14B—H14B	0.9300
N12A—H12B	0.80 (5)	C15B—C16B	1.359 (5)
N13A—C18A	1.328 (3)	C15B—H15B	0.9300
N13A—C14A	1.344 (3)	C16B—C17B	1.366 (5)
C14A—C15A	1.369 (4)	C16B—H16B	0.9300
C14A—H14A	0.9300	C17B—C18B	1.388 (4)
C15A—C16A	1.377 (6)	C17B—H17B	0.9300
C15A—H15A	0.9300	C18B—H18B	0.9300
			
O1A—Zn1—O1B	76.54 (6)	C16A—C15A—H15A	120.5
O1A—Zn1—N13A	101.47 (7)	C17A—C16A—C15A	119.6 (3)
O1B—Zn1—N13A	102.02 (7)	C17A—C16A—H16A	120.2
O1A—Zn1—N8A	86.63 (6)	C15A—C16A—H16A	120.2
O1B—Zn1—N8A	149.97 (7)	C16A—C17A—C18A	118.4 (4)
N13A—Zn1—N8A	105.63 (7)	C16A—C17A—H17A	120.8
O1A—Zn1—S11A	150.62 (5)	C18A—C17A—H17A	120.8
O1B—Zn1—S11A	100.35 (5)	N13A—C18A—C17A	123.0 (3)
N13A—Zn1—S11A	107.69 (6)	N13A—C18A—H18A	118.5
N8A—Zn1—S11A	82.28 (5)	C17A—C18A—H18A	118.5
O1B—Zn2—O1A	76.14 (6)	C1B—O1B—Zn2	125.18 (13)
O1B—Zn2—N8B	86.44 (6)	C1B—O1B—Zn1	130.98 (13)
O1A—Zn2—N8B	152.37 (7)	Zn2—O1B—Zn1	103.30 (6)
O1B—Zn2—N13B	100.18 (7)	O1B—C1B—C6B	120.23 (19)
O1A—Zn2—N13B	103.89 (7)	O1B—C1B—C2B	121.60 (19)
N8B—Zn2—N13B	100.15 (7)	C6B—C1B—C2B	118.17 (19)
O1B—Zn2—S11B	150.41 (5)	C5B—C6B—C1B	122.2 (2)
O1A—Zn2—S11B	100.87 (4)	C5B—C6B—H6B	118.9
N8B—Zn2—S11B	83.77 (5)	C1B—C6B—H6B	118.9
N13B—Zn2—S11B	108.99 (6)	C4B—C5B—C6B	119.9 (2)
C1A—O1A—Zn1	130.37 (13)	C4B—C5B—H5B	120.1
C1A—O1A—Zn2	124.97 (12)	C6B—C5B—H5B	120.1
Zn1—O1A—Zn2	102.89 (6)	C3B—C4B—C5B	119.0 (2)
O1A—C1A—C6A	119.5 (2)	C3B—C4B—H4B	120.5
O1A—C1A—C2A	121.95 (19)	C5B—C4B—H4B	120.5
C6A—C1A—C2A	118.6 (2)	C4B—C3B—C2B	122.6 (2)
C5A—C6A—C1A	121.7 (2)	C4B—C3B—H3B	118.7
C5A—C6A—H6A	119.2	C2B—C3B—H3B	118.7
C1A—C6A—H6A	119.2	C3B—C2B—C1B	118.2 (2)
C4A—C5A—C6A	120.0 (2)	C3B—C2B—C7B	116.8 (2)
C4A—C5A—H5A	120.0	C1B—C2B—C7B	124.94 (19)
C6A—C5A—H5A	120.0	N8B—C7B—C2B	125.3 (2)
C3A—C4A—C5A	119.4 (2)	N8B—C7B—H7B	117.4
C3A—C4A—H4A	120.3	C2B—C7B—H7B	117.4
C5A—C4A—H4A	120.3	C7B—N8B—N9B	115.74 (18)
C4A—C3A—C2A	122.2 (2)	C7B—N8B—Zn2	124.19 (15)
C4A—C3A—H3A	118.9	N9B—N8B—Zn2	119.54 (13)
C2A—C3A—H3A	118.9	C10B—N9B—N8B	113.85 (18)
C3A—C2A—C1A	118.0 (2)	N9B—C10B—N12B	116.7 (2)
C3A—C2A—C7A	117.0 (2)	N9B—C10B—S11B	127.55 (17)
C1A—C2A—C7A	124.97 (19)	N12B—C10B—S11B	115.73 (18)
N8A—C7A—C2A	125.6 (2)	C10B—S11B—Zn2	94.66 (7)
N8A—C7A—H7A	117.2	C10B—N12B—H12C	117 (2)
C2A—C7A—H7A	117.2	C10B—N12B—H12D	118 (2)
C7A—N8A—N9A	114.98 (18)	H12C—N12B—H12D	124 (3)
C7A—N8A—Zn1	127.86 (15)	C18B—N13B—C14B	118.1 (2)
N9A—N8A—Zn1	116.97 (14)	C18B—N13B—Zn2	119.74 (17)
C10A—N9A—N8A	113.92 (19)	C14B—N13B—Zn2	121.90 (17)
N9A—C10A—N12A	116.6 (3)	N13B—C14B—C15B	122.3 (3)
N9A—C10A—S11A	126.55 (18)	N13B—C14B—H14B	118.8
N12A—C10A—S11A	116.9 (2)	C15B—C14B—H14B	118.8
C10A—S11A—Zn1	92.79 (9)	C16B—C15B—C14B	119.2 (3)
C10A—N12A—H12A	116 (3)	C16B—C15B—H15B	120.4
C10A—N12A—H12B	116 (4)	C14B—C15B—H15B	120.4
H12A—N12A—H12B	120 (5)	C15B—C16B—C17B	119.4 (3)
C18A—N13A—C14A	118.3 (2)	C15B—C16B—H16B	120.3
C18A—N13A—Zn1	119.36 (17)	C17B—C16B—H16B	120.3
C14A—N13A—Zn1	121.76 (19)	C16B—C17B—C18B	118.7 (3)
N13A—C14A—C15A	121.7 (3)	C16B—C17B—H17B	120.7
N13A—C14A—H14A	119.2	C18B—C17B—H17B	120.7
C15A—C14A—H14A	119.2	N13B—C18B—C17B	122.3 (3)
C14A—C15A—C16A	119.0 (3)	N13B—C18B—H18B	118.9
C14A—C15A—H15A	120.5	C17B—C18B—H18B	118.9
			
O1B—Zn1—O1A—C1A	−173.10 (19)	O1A—Zn2—O1B—C1B	164.28 (19)
N13A—Zn1—O1A—C1A	87.11 (19)	N8B—Zn2—O1B—C1B	−37.35 (18)
N8A—Zn1—O1A—C1A	−18.16 (18)	N13B—Zn2—O1B—C1B	62.33 (19)
S11A—Zn1—O1A—C1A	−85.9 (2)	S11B—Zn2—O1B—C1B	−108.09 (18)
O1B—Zn1—O1A—Zn2	−8.04 (7)	O1A—Zn2—O1B—Zn1	−8.06 (7)
N13A—Zn1—O1A—Zn2	−107.83 (8)	N8B—Zn2—O1B—Zn1	150.32 (9)
N8A—Zn1—O1A—Zn2	146.89 (8)	N13B—Zn2—O1B—Zn1	−110.00 (8)
S11A—Zn1—O1A—Zn2	79.16 (10)	S11B—Zn2—O1B—Zn1	79.58 (11)
O1B—Zn2—O1A—C1A	174.33 (18)	O1A—Zn1—O1B—C1B	−163.4 (2)
N8B—Zn2—O1A—C1A	121.86 (18)	N13A—Zn1—O1B—C1B	−64.3 (2)
N13B—Zn2—O1A—C1A	−88.40 (17)	N8A—Zn1—O1B—C1B	138.90 (19)
S11B—Zn2—O1A—C1A	24.48 (17)	S11A—Zn1—O1B—C1B	46.4 (2)
O1B—Zn2—O1A—Zn1	8.20 (7)	O1A—Zn1—O1B—Zn2	8.26 (7)
N8B—Zn2—O1A—Zn1	−44.27 (17)	N13A—Zn1—O1B—Zn2	107.35 (8)
N13B—Zn2—O1A—Zn1	105.47 (8)	N8A—Zn1—O1B—Zn2	−49.41 (17)
S11B—Zn2—O1A—Zn1	−141.65 (5)	S11A—Zn1—O1B—Zn2	−141.87 (6)
Zn1—O1A—C1A—C6A	−160.87 (18)	Zn2—O1B—C1B—C6B	−152.5 (2)
Zn2—O1A—C1A—C6A	37.0 (3)	Zn1—O1B—C1B—C6B	17.6 (3)
Zn1—O1A—C1A—C2A	19.1 (3)	Zn2—O1B—C1B—C2B	28.1 (3)
Zn2—O1A—C1A—C2A	−143.01 (16)	Zn1—O1B—C1B—C2B	−161.84 (17)
O1A—C1A—C6A—C5A	−176.8 (3)	O1B—C1B—C6B—C5B	178.0 (3)
C2A—C1A—C6A—C5A	3.2 (4)	C2B—C1B—C6B—C5B	−2.5 (4)
C1A—C6A—C5A—C4A	−1.7 (5)	C1B—C6B—C5B—C4B	0.9 (5)
C6A—C5A—C4A—C3A	−0.9 (5)	C6B—C5B—C4B—C3B	1.0 (6)
C5A—C4A—C3A—C2A	2.0 (5)	C5B—C4B—C3B—C2B	−1.3 (5)
C4A—C3A—C2A—C1A	−0.4 (4)	C4B—C3B—C2B—C1B	−0.3 (4)
C4A—C3A—C2A—C7A	−178.6 (3)	C4B—C3B—C2B—C7B	177.6 (3)
O1A—C1A—C2A—C3A	177.8 (2)	O1B—C1B—C2B—C3B	−178.4 (2)
C6A—C1A—C2A—C3A	−2.2 (3)	C6B—C1B—C2B—C3B	2.2 (4)
O1A—C1A—C2A—C7A	−4.1 (3)	O1B—C1B—C2B—C7B	3.9 (4)
C6A—C1A—C2A—C7A	175.9 (2)	C6B—C1B—C2B—C7B	−175.6 (3)
C3A—C2A—C7A—N8A	172.1 (2)	C3B—C2B—C7B—N8B	169.7 (3)
C1A—C2A—C7A—N8A	−6.0 (4)	C1B—C2B—C7B—N8B	−12.5 (4)
C2A—C7A—N8A—N9A	−173.8 (2)	C2B—C7B—N8B—N9B	177.1 (2)
C2A—C7A—N8A—Zn1	1.1 (3)	C2B—C7B—N8B—Zn2	−11.3 (4)
O1A—Zn1—N8A—C7A	7.88 (19)	O1B—Zn2—N8B—C7B	28.7 (2)
O1B—Zn1—N8A—C7A	63.3 (3)	O1A—Zn2—N8B—C7B	79.2 (3)
N13A—Zn1—N8A—C7A	−93.1 (2)	N13B—Zn2—N8B—C7B	−71.0 (2)
S11A—Zn1—N8A—C7A	160.6 (2)	S11B—Zn2—N8B—C7B	−179.3 (2)
O1A—Zn1—N8A—N9A	−177.33 (17)	O1B—Zn2—N8B—N9B	−160.02 (17)
O1B—Zn1—N8A—N9A	−121.94 (17)	O1A—Zn2—N8B—N9B	−109.53 (19)
N13A—Zn1—N8A—N9A	81.69 (17)	N13B—Zn2—N8B—N9B	100.26 (17)
S11A—Zn1—N8A—N9A	−24.60 (16)	S11B—Zn2—N8B—N9B	−7.98 (16)
C7A—N8A—N9A—C10A	−167.0 (2)	C7B—N8B—N9B—C10B	−179.8 (2)
Zn1—N8A—N9A—C10A	17.6 (3)	Zn2—N8B—N9B—C10B	8.2 (3)
N8A—N9A—C10A—N12A	−175.6 (3)	N8B—N9B—C10B—N12B	176.0 (2)
N8A—N9A—C10A—S11A	6.1 (4)	N8B—N9B—C10B—S11B	−3.0 (3)
N9A—C10A—S11A—Zn1	−21.8 (3)	N9B—C10B—S11B—Zn2	−2.6 (2)
N12A—C10A—S11A—Zn1	159.9 (3)	N12B—C10B—S11B—Zn2	178.4 (2)
O1A—Zn1—S11A—C10A	88.84 (13)	O1B—Zn2—S11B—C10B	76.08 (12)
O1B—Zn1—S11A—C10A	169.75 (11)	O1A—Zn2—S11B—C10B	157.12 (9)
N13A—Zn1—S11A—C10A	−83.96 (11)	N8B—Zn2—S11B—C10B	4.67 (9)
N8A—Zn1—S11A—C10A	20.05 (11)	N13B—Zn2—S11B—C10B	−93.94 (10)
O1A—Zn1—N13A—C18A	11.1 (2)	O1B—Zn2—N13B—C18B	−166.50 (17)
O1B—Zn1—N13A—C18A	−67.4 (2)	O1A—Zn2—N13B—C18B	115.40 (17)
N8A—Zn1—N13A—C18A	100.8 (2)	N8B—Zn2—N13B—C18B	−78.33 (18)
S11A—Zn1—N13A—C18A	−172.51 (18)	S11B—Zn2—N13B—C18B	8.51 (18)
O1A—Zn1—N13A—C14A	−177.97 (19)	O1B—Zn2—N13B—C14B	7.98 (19)
O1B—Zn1—N13A—C14A	103.55 (19)	O1A—Zn2—N13B—C14B	−70.11 (19)
N8A—Zn1—N13A—C14A	−88.3 (2)	N8B—Zn2—N13B—C14B	96.16 (18)
S11A—Zn1—N13A—C14A	−1.6 (2)	S11B—Zn2—N13B—C14B	−177.00 (17)
C18A—N13A—C14A—C15A	2.2 (4)	C18B—N13B—C14B—C15B	−0.5 (4)
Zn1—N13A—C14A—C15A	−168.9 (2)	Zn2—N13B—C14B—C15B	−175.1 (2)
N13A—C14A—C15A—C16A	0.4 (5)	N13B—C14B—C15B—C16B	1.4 (4)
C14A—C15A—C16A—C17A	−1.9 (6)	C14B—C15B—C16B—C17B	−1.4 (5)
C15A—C16A—C17A—C18A	0.9 (6)	C15B—C16B—C17B—C18B	0.7 (5)
C14A—N13A—C18A—C17A	−3.3 (4)	C14B—N13B—C18B—C17B	−0.2 (4)
Zn1—N13A—C18A—C17A	167.9 (3)	Zn2—N13B—C18B—C17B	174.5 (2)
C16A—C17A—C18A—N13A	1.8 (5)	C16B—C17B—C18B—N13B	0.1 (4)

### Hydrogen-bond geometry (Å, °)

**Table:**

*D*—H···*A*	*D*—H	H···*A*	*D*···*A*	*D*—H···*A*
N12A—H12B···N9B^i^	0.80 (5)	2.40 (5)	3.195 (4)	174 (6)
N12B—H12C···S11A^ii^	0.77 (3)	2.74 (3)	3.510 (3)	177 (3)
N12B—H12D···N9A^iii^	0.88 (3)	2.14 (3)	3.012 (3)	173 (3)
C17B—H17B···S11A^iv^	0.93	2.91	3.772 (3)	156

Symmetry codes: (i) *x*−1, −*y*+1/2, *z*−1/2; (ii) *x*+1, *y*, *z*; (iii) *x*+1, −*y*+1/2, *z*+1/2; (iv) −*x*+2, *y*−1/2, −*z*+3/2.
